# Current Status of Management of Hepatocellular Carcinoma in The Gulf Region: Challenges and Recommendations

**DOI:** 10.3390/cancers15072001

**Published:** 2023-03-28

**Authors:** Jasem Albarrak, Humaid Al-Shamsi

**Affiliations:** 1 Kuwait Cancer Control Center, Sabah Health Region, Kuwait City 8WF3+WR8, Kuwait; jaalbarrak@moh.gov.kw; 2 Burjeel Medical City- Burjeel Holding, Abu Dhabi 92510, United Arab Emirates; 3 College of Medicine, University of Sharjah, Sharjah 27272, United Arab Emirates; 4 Emirates Oncology Society, Dubai 22107, United Arab Emirates

**Keywords:** Gulf region, hepatocellular carcinoma, immune-checkpoint inhibitors, locoregional therapy, management of HCC, systemic therapy

## Abstract

**Simple Summary:**

The incidence of liver cancer is rising globally and is estimated to reach >1 million cases by 2025. Hepatocellular carcinoma (HCC) accounts for ~90% of the cases of liver cancer and is associated with a high healthcare expenditure and death rate. The most prominent risk factors for HCC include hepatitis B and C viral infections and non-alcoholic steatohepatitis associated with metabolic syndrome or type 2 diabetes. There has been a steady increase in the diagnosis of liver cancer in the Arabian Gulf region, possibly due to the high incidence of obesity, diabetes, and viral hepatitis. The diversity of the Gulf population makes it imperative to develop and implement effective screening programs for the early diagnosis and treatment of HCC. In this review, we discuss the available literature on the epidemiology, screening, and management of HCC in the Gulf region.

**Abstract:**

The burden of hepatocellular carcinoma (HCC) is on the rise in the Gulf region, with most patients being diagnosed in the intermediate or advanced stages. Surgery is a treatment option for only a few, and the majority of patients receive either locoregional treatment (percutaneous ethanol injection, radiofrequency ablation, transarterial chemoembolization [TACE], radioembolization, radiotherapy, or transarterial radioembolization) or systemic therapy (for those ineligible for locoregional treatments or who do not benefit from TACE). The recent emergence of novel immunotherapies such as immune checkpoint inhibitors has begun to change the landscape of systemic HCC treatment in the Gulf. The combination of atezolizumab and bevacizumab is currently the preferred first-line therapy in patients not at risk of bleeding. Additionally, the HIMALAYA trial has demonstrated the superiority of the durvalumab plus tremelimumab combination (STRIDE regimen) therapy in efficacy and safety compared with sorafenib in patients with unresectable HCC. However, there is a lack of data on post-progression treatment after first-line therapy with either atezolizumab plus bevacizumab or durvalumab plus tremelimumab regimens, highlighting the need for better-designed studies for improved management of patients with unresectable HCC in the Gulf region.

## 1. Introduction

Liver cancer is a growing global health challenge, with an estimated annual incidence of >1 million cases worldwide by 2025 [1,2,3,4]. Hepatocellular carcinoma (HCC) accounts for approximately 90% of the cases of liver cancer and is associated with high morbidity and mortality [1,5]. The incidence of HCC shows a strong male preponderance, ranking third as a cause of cancer-related deaths in males, and increases progressively with advancing age in all populations [5,6]. The most prominent risk factors for HCC include hepatitis B (HBV) and hepatitis C (HCV) viral infections and non-alcoholic steatohepatitis (NASH), which can lead to liver fibrosis and cirrhosis [1,6,7,8,9,10]. Other risk factors include heavy alcohol consumption, non-alcoholic fatty liver disease (NAFLD), consumption of aflatoxins, obesity associated with metabolic syndrome, type 2 diabetes (T2D), and tobacco smoking [11]. The epidemiology of these risk factors depends on geographic location and host-specific factors.

The Arabian Gulf region encompasses six countries—Bahrain, Kuwait, Oman, Qatar, Saudi Arabia, and the United Arab Emirates (UAE)—which together constitute the Gulf Cooperation Council (GCC) [12]. The GCC countries are marked by the diversity of their resident populations and exhibit one of the highest ratios of migrants to nationals in the world. More than half of the regional population is expatriate, with non-nationals comprising more than three-quarters of the populations in Kuwait, Qatar, and the UAE [12,13,14]. There has been a steady increase in the diagnosis of liver cancer in the GCC region, representing 5.2% of all cancers diagnosed between 1997 and 2007, possibly explained by the increase in population growth [12,13,14]. 

Early diagnosis and effective treatment of HCC remain a challenge, with some patients showing symptoms of right upper abdominal quadrant pain, anorexia, early satiety, weight loss, obstructive jaundice, fever, watery diarrhea, lethargy, and bone pain while the majority remain asymptomatic. Consequently, most patients present with an advanced stage of the disease [11]. The etiological differences among the population affected with HCC in the Gulf region make it imperative to develop and implement effective screening programs for early diagnosis and treatment to improve patient outcomes [13,14,15,16,17,18]. In this review, we discuss the available literature on the epidemiological factors, clinicopathological characteristics, screening practices, and management strategies for intermediate- and advanced-stage HCC in the Gulf region. This article sets the foundation for analyzing gaps and providing recommendations for practicing physicians, regulators, stakeholders, and decision-makers to improve the screening and management of unresectable HCC in the GCC region.

## 2. Epidemiology of HCC across the Gulf Region

**Figure 1:**
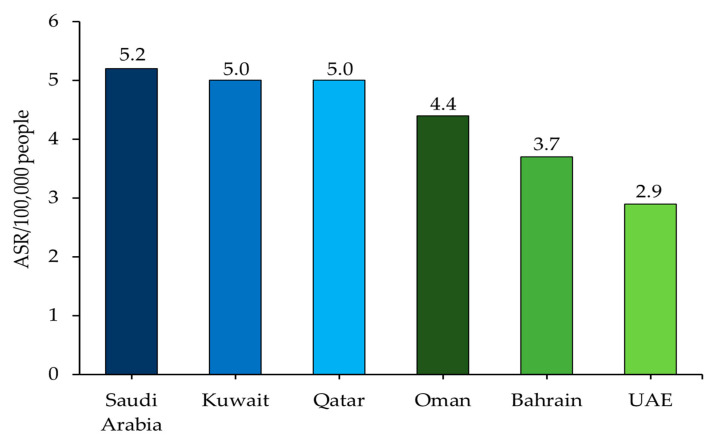
HCC incidence rates in the GCC countries: GLOBOCAN 2020. ASR, age-standardized incidence rate; GCC, Gulf Cooperation Council; HCC, hepatocellular carcinoma; UAE, United Arab Emirates.

The GCC region comprises a population of around 60 million, with very large expatriate communities [19]. Between 1998 and 2012, a total of 8012 liver cancer cases were reported from all GCC states, comprising 4.9% of all the cancer cases recorded [20]. The majority of cases were reported from Saudi Arabia (83.4%), followed by Oman (6.6%), Kuwait (4.3%), Bahrain (2.4%), the UAE (1.8%), and Qatar (1.6%) [20]. The age-standardized incidence rate (ASR) of HCC in the region, as per the latest report by the World Health Organization (WHO), is 4.7 per 100,000 people with a mortality rate of 4.5 (Figure 1) [18,21,22,23,24,25,26]. From 2001 to 2014, temporal trends indicated a rising incidence of primary hepatic carcinoma in Saudi Arabia. In 2014, the Saudi Cancer Registry announced that liver cancer ranked sixth among Saudi males and ninth among Saudi females in terms of cancer incidence [27]. HCC (79.3%) comprised the majority of liver cancer cases, followed by cholangiocarcinoma (11%), and hepatoblastoma (4.7%), with a significantly higher incidence among males [28]. Sharafi and Alavian reported that Qatar had the highest liver cancer mortality rate among GCC member countries in 2017 [23]. A study on 180 patients with HCC from Qatar between 2011 and 2015 showed HCC incidence to be on the rise, with tumors >5 cm and bilobular involvement leading to extrahepatic metastasis [29]. According to the Cancer Registry in Oman, the ASR for liver cancer in males increased from 4.7 in 2007 to 6.3 in 2019, with the highest incidence rate of 10.2 recorded in 2016. The ASR was lower in Omani females, increasing from 1.2 in 2007 to 2.6 in 2019, with the highest incidence rate of 4.8 recorded in 2016 [30]. In a retrospective case-series study conducted between January 2008 and December 2015 at the three main tertiary care hospitals in Oman, the majority of patients with HCC were male with liver cirrhosis due to viral hepatitis. Additionally, few patients presented with advanced disease, precluding therapeutic or even palliative treatment [31]. In 2019, a total of 68 cases of liver cancer were recorded in Oman, with 46 hospital deaths [30]. The incidence rate of HCC in UAE has moved up from the 17th position in the 1980s to the 13th position recently within the Middle East and Africa (MEA) region, with the crude incidence rate being 0.8 per 100,000 people in 2017 [32,33]. The incidence rate of HCC was almost double in males (47 of 72 reported cases in 2017) versus females (25 of 72 reported cases in 2017) in the UAE, thus conforming to the global trend [32,33].

The rising HCC burden in the GCC region may be due to the increasing rates of obesity and T2D, an aging population, and an increasing prevalence of viral hepatitis [33]. Obesity induces metabolic syndrome and inflammation and is an etiological factor for NAFLD, NASH, hepatic fibrosis, cirrhosis, and ultimately HCC. Studies have shown that a high body mass index, waist circumference, and T2D are associated with higher risks of HCC [11]. Possible mechanisms of T2D-associated increases in the risk of HCC may include indirect activation of insulin-like growth factor pathways, altering endogenous sex hormones, or chronic inflammation [14,34,35]. According to the most recent WHO reports, Egypt, Bahrain, Kuwait, Oman Saudi Arabia, and the UAE are among the ten countries in the world with the highest diabetes and prediabetes prevalence [13,14,15,17]. A recent retrospective study from a single center in Kuwait showed more than a third of the screened patients had T2D as a risk factor for HCC [14]. In another recent predictive modeling study on adults with obesity and T2D from Saudi Arabia and the UAE, prevalent cases of compensated cirrhosis and advanced liver disease were projected to double by 2030 [36]. NAFLD and NASH, associated with obesity and T2D, are common problems among the native resident population of the Gulf [13,16,17]. Morbidity related to NAFLD and its resulting NASH has dramatically increased in the last decade, especially in the Gulf countries, where there is already an epidemic of obesity and T2D, including pediatric and adolescent obesity. The high prevalence of obesity and T2D is likely to cause a further increase in NAFLD morbidity and NASH-associated advanced liver disease in the coming decades [36,37,38,39,40]. In Saudi Arabia, it is estimated that approximately 12 million individuals will be diagnosed with NAFLD by 2030 [36]. Similarly, the cases of advanced liver disease secondary to NAFLD, NASH, and HCC are projected to double by 2030, with an annual incidence of liver-related deaths of 4800 [36]. NASH is likely to be the leading cause of liver transplantation in the Gulf countries due to a reduced burden of viral hepatitis among the resident nationals, in combination with skyrocketing obesity rates [36,41,42].

Infection with HCV is a significant cause of chronic hepatitis, cirrhosis, and HCC and is the leading cause of liver transplantation globally [12,43,44]. HCV promotes cellular proliferation, steatosis, inflammatory processes, mitochondrial dysfunction, and insulin resistance, all leading to oxidative stress, genetic instability, and DNA damage, with liver cirrhosis and HCC as the likely outcomes [11,45]. The MEA regions have the highest global prevalence of HCV infection, with Egypt being the most affected [12,43,44,46,47,48,49,50]. Although the prevalence of HCV infections varies significantly among the GCC member states, the overall national-level HCV prevalence in the GCC region was found to be comparable to global levels [47,48]. The prevalence rate of HCV among nationals tends to be lower than among resident expatriates, with a higher prevalence found in specific expatriate populations such as Egyptians, reflecting the prevalence in their countries of origin [49,50]. Additionally, the prevalence of HBV infection in the MEA region (0.6–8%) is also higher than in Western Europe and North America (<1%), with the rate of infection being >4% among the GCC states [47,49,51,52,53,54]. Apparent HBV prevalence in the GCC region ranges from 0–3% in the UAE, 2–4% in Qatar, 0.5–5% in Kuwait, 3–4% in Saudi Arabia, 0–6% in Bahrain, and 2–6% in Oman [49,50,54,55]. HBV-induced pathogenesis of HCC involves several mechanisms, including HBV-DNA integration into host genetic machinery, DNA methylation, oxidative stress, and HBx protein [11,45]. Individuals with chronic HBV infection are at higher risk of developing end-stage liver disease, including cirrhosis, liver failure, and HCC [49,50,51,53]. The prevalence of HBV infection has decreased in recent years in the younger population of the Gulf region due to the vaccination program [56]. However, the prevalence of HBV has not been well-characterized in the older population and remains a source of concern [56]. 

Co-infection with HBV and HCV has a worse prognosis than infection with either virus alone and is associated with more comorbidities, increased severity of liver disease, and a higher risk of HCC [47,49,57,58]. HBV and HCV viral proteins are involved in hijacking the cellular machinery and causing cirrhotic tissue development through the release of proinflammatory cytokines like interleukin (IL)6, tumor necrosis factor (TNF)-α, IL1, and IL18 [11,45]. The global burden of viral-associated HCC, analyzed for the 22 Middle Eastern countries by age, sex, and economic status from 1990 to 2010, revealed that 77% of deaths due to HCC were HBV/HCV-linked. From 1990 to 2010, the burden of HBV and HCV-associated HCC deaths in the Arab world increased by 137% and 216%, respectively, as compared with global increases of 62% and 73%, respectively, indicating a much faster rate of increase in HBV/HCV-associated HCC in the Middle East than the rest of the world. Male sex and low economic status correlated with higher rates [59]. For the GCC member countries, a rising population of expatriates compared to nationals may be responsible for the increasing incidence of viral-associated HCC in the region [12,50,52,57]. Two retrospective studies from Kuwait and Qatar showed the rate of HCV-related HCC among the resident expatriate populations to be almost twice that of the resident nationals [49,60]. Recent data from the Cancer Registry of UAE have shown the incidence of HCC in non-UAE citizens has increased from almost twice in 2017 to almost thrice that of UAE citizens in 2019 [61,62]. The fact that HCC incidence is more common in males than females generally reflects the population structure of the Gulf countries, especially Kuwait and Qatar, where there are more males than females due to the presence of expatriate workers [13,14,15,17,63,64]. Among the resident nationals, the difference in HCC incidence between males and females is much less evident [13,14,15,17,63,64]. The gradually increasing number of cases of HCC in the GCC region has put additional demands on the healthcare system, especially for clinical oncology services, particularly since most patients present with advanced-stage HCC. Early diagnosis involving routine screening protocols in high-risk patients is crucial for effective treatment.

## 3. HCC Management in the Gulf: Staging and Treatment Modalities for Unresectable Intermediate and Advanced HCC

**Figure 2:**
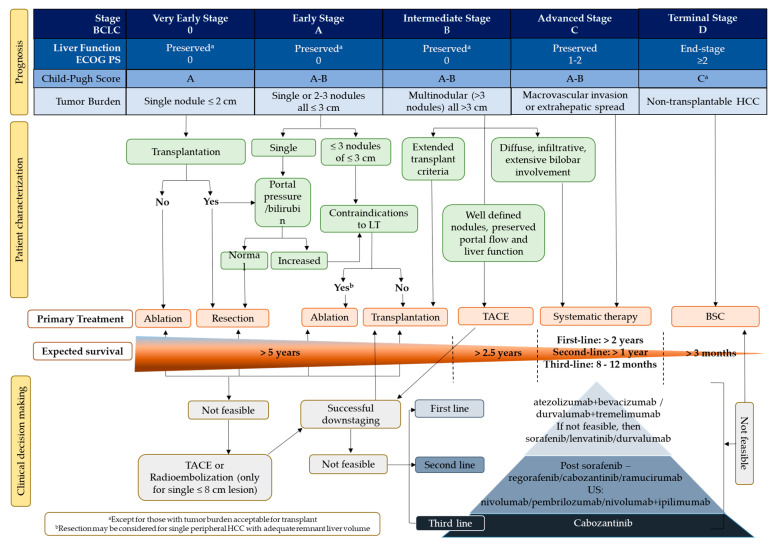
HCC treatment landscape. BCLC, Barcelona Clinic Liver Cancer; BSC, best supportive care; ECOG PS, Eastern Cooperative Oncology Group performance status; HCC, hepatocellular carcinoma; LT, liver transplant; TACE, transarterial chemoembolization; US, United States.

HCC is unique in its association with chronic liver disease, which may sequentially progress to fibrosis and then to cirrhosis, eventually culminating in neoplasia [54]. The prognosis of individual patients with HCC is dependent not only on the etiology of the tumor but also on the degree of functional failure of the liver due to the presence of cirrhosis [56]. The role of chronic liver disease in the prognosis of HCC is evidenced by the inclusion of the Child-Pugh score—a three-category scale (A, B, and C), with C indicating the most severe compromise of liver function—or other aspects linked to liver functions in several staging systems used for HCC. A compensated liver function could be further stratified by using the albumin-bilirubin (ALBI) score and the alpha-fetoprotein (AFP) concentration, irrespective of tumor burden [57,58,60,61,62]. Depending on the stage and severity of the disease, HCC can be classified as resectable, transplantable, unresectable, or metastatic [56,61,63,64,65,66,67]. The 2022 Barcelona Clinic Liver Cancer (BCLC) staging and treatment strategy highlighted the different concepts and parameters that physicians and multidisciplinary tumor boards should integrate for a personalized approach to treating HCC (Figure 2) [68]. Since most patients with HCC are diagnosed at the intermediate or advanced stage, there are limited curative therapeutic options [64]. Surgery is a treatment option for only a limited number of patients. Most patients received locoregional treatment or systemic therapy. Locoregional treatment options include percutaneous ethanol injection, radiofrequency ablation (RFA), transarterial chemoembolization (TACE), radioembolization, and radiotherapy [1,13,69]. Transarterial radioembolization is considered in patients with single nodules of ≤8 cm for whom surgical resection or ablation are not feasible options. In patients who are not eligible for locoregional treatments, including those with unresectable advanced-stage disease, systemic therapy is recommended as the first choice of treatment [62]. Patients with HCC categorized as BCLC-B are further classified into three groups according to tumor burden and liver function. The first subgroup within BCLC-B includes patients with well-defined HCC nodules who would be eligible for liver transplants [68,70]. The second subgroup comprises patients with preserved portal flow and a defined tumor burden who are ineligible for liver transplantation possibly due to selective access to feeding tumor arteries. These patients are candidates for TACE unless they are found ineligible, in which case systemic therapy is considered [66,68]. The third subgroup within BCLC-B comprises patients with diffuse, infiltrative, and extensive HCC who do not benefit from TACE; systemic therapy is the recommended option, although there is no strict cutoff for when this may be initiated [68].

### 3.1. Surgery for Patients with HCC across the Gulf

The Milan criteria (one lesion ≥2 cm to ≤5 cm, or up to three lesions, each ≥1 cm to ≤3 cm) are still largely applied to select patients with HCC for liver transplantation in the Gulf [70,71,72]. HCC tumors are multifocal. The diagnosis is strongly associated with vascular invasion. Portal or hepatic vein infiltration can predict tumor recurrence after liver transplantation [13,73]. Most patients (61.2%) from a single center in Kuwait demonstrated multifocal tumors at the time of diagnosis, with surgical resection done in only a small proportion of them (8.3%) [13,14]. However, there is no consensus on expanded criteria for liver transplantation in patients with HCC [13,14]. In a small proportion of patients with unresectable HCC, salvage surgery after successful tumor downstaging can provide long-term control of the disease [73,74,75].

### 3.2. Role of Locoregional Therapy for Patients with HCC across the Gulf

Locoregional treatments for intermediate and advanced-stage (BCLC-B and BCLC-C) unresectable HCC are aimed at eliminating or reducing tumor viability, delaying progression, and ultimately extending overall survival (OS) with preservation of liver function [76,77]. Guidelines recommend TACE for patients with intermediate-stage HCC, leading to a median survival of ≥2.5 years [6,69,78,79]. TACE can also be applied to unresectable early-stage (BCLC-A) as well as advanced-stage (BCLC-C) patients with HCC. However, for patients to be eligible for TACE, liver function needs to be well preserved. Assessing the effect of TACE on liver function is difficult, with only a limited proportion of patients suffering deterioration [80]. Clinically meaningful thresholds of worsening in laboratory values after TACE have been used recently. Increased bilirubin (>2 mg/dL) or slight fluid retention requiring diuretic treatment are associated with an increased risk of adverse events (AEs) and suboptimal survival after TACE [78]. In a recent cohort study of 70 patients treated with a superselective conventional TACE, a post procedure increase of transaminases (aspartate transaminase [AST] ≥46%, alanine transaminase [ALT] ≥ 52%) compared with baseline values was shown to be a reliable predictor of overall objective response in clinical practice, although not associated with liver function deterioration [80]. 

**Figure 3:**
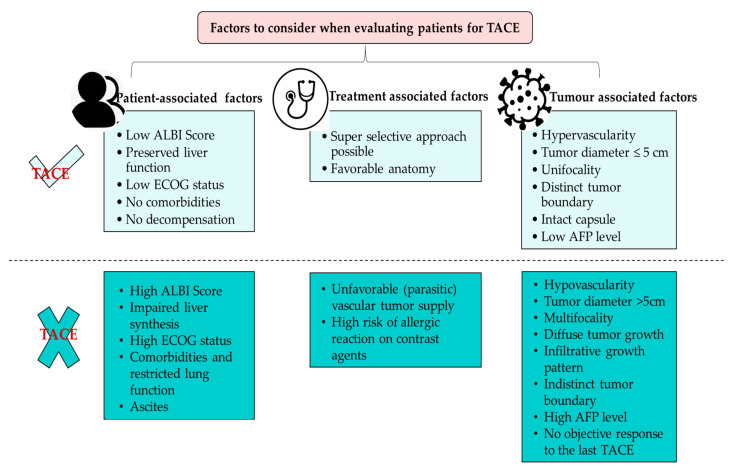
Overview of prognostic factors for patient selection for TACE. Figure adapted from Müller L et al., JHC2021 [84]. AFP, alphaa-fetoprotein; ALBI, albumin-bilirubin; ECOG, Eastern Cooperative Oncology Group; TACE, transarterial chemoembolization.

A retrospective analysis of 150 patients with HCC in Qatar found an improved median survival of 27 months (95% CI 20.3–33.7) in patients who received TACE as first-line treatment [15]. In another retrospective study from a single center in Kuwait, 12.6% of the patients underwent TACE, while RFA was done in 8.1% of the patients [13]. A study in Japan demonstrated that TACE could be done three times at intervals of 3 months with an OS of 21 months. However, the ALBI score deteriorated in patients receiving repeat TACE [81]. TACE is not very effective in many patients in the Gulf with intermediate-stage HCC due to the heterogeneity in tumor burden and liver function. According to the Asia-Pacific Primary Liver Cancer Expert Consensus Statement 2019, TACE is not beneficial for three subgroups of patients: (i) patients who easily become refractory to TACE; (ii) patients in whom TACE causes deterioration of hepatic functional reserve to Child-Pugh class B; and (iii) patients who are unlikely to respond to TACE (TACE-resistant tumor) [57,81]. Thus, identifying the eligible population that will benefit more from repeat treatment with TACE compared with those eligible for systemic therapy is of vital importance (Figure 3) [51,77,81,82,83,84,85]. Several scores have been developed for identifying suitable candidates for repeat TACE considering the changes in functional liver variables before and after TACE. These scores were mainly designed to stratify candidates by anticipated survival, similar to those designed for identifying candidates for the first TACE [86]. Large studies specifically analyzing the effect of repeated TACE on liver functional reserve are still lacking. Even a subclinical deterioration of liver function after repeat TACE may lead to the transition from Child-Pugh class A to B cirrhosis, thereby preventing the patient from receiving effective systemic therapies and thus possibly achieving a complete tumor response [86].

**Figure 4:**
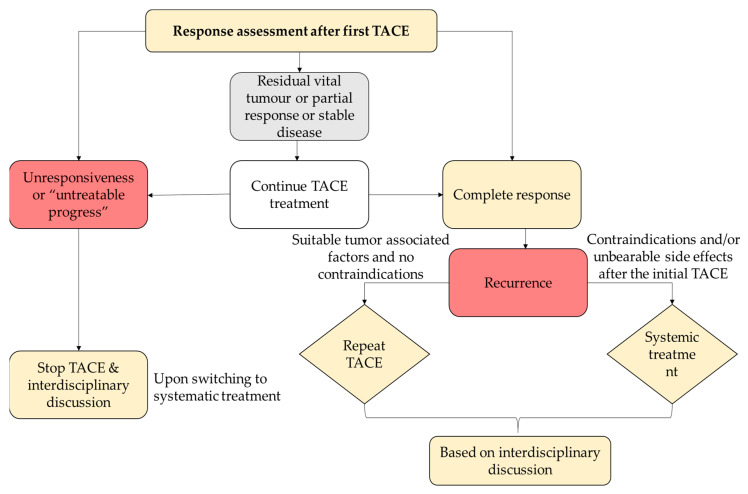
Re-evaluation criteria and decision-making during TACE. Figure adapted from Müller L et al., JHC2021 [84]. TACE: transarterial chemoembolization.

Re-evaluation of patients’ responses after the initial TACE procedure is an indispensable factor for further guidance, as it functions as a strong predictor of median OS [84]. As tumor necrosis is not necessarily accompanied by an immediate reduction of the tumor size, conventional Response Evaluation Criteria in Solid Tumors (RECIST) criteria based on the maximum tumor diameter may tend to underestimate the tumor response [84]. Modified RECIST criteria measuring the sum of the longest diameter of contrast-enhancing tissue have been developed and are the recommended assessment of response by Western guidelines [87]. However, as in the conventional criteria, the patient’s response to TACE can be classified as a complete response, a partial response, stable disease, or progressive disease (Figure 4) [84].

### 3.3. Systemic Therapies Used for Patients with HCC across the Gulf

The recent emergence of novel immunotherapies such as immune checkpoint inhibitors (ICIs) as monotherapy or combination treatments with other drug targets has begun to change the landscape of systemic HCC treatment in the Gulf [82,87,88]. Since 2020, following the positive safety and efficacy findings from the Phase III IMbrave150 trial, there has been a shift in the paradigm of the practice in the GCC region [89]. The combination of the programmed cell death (PD) ligand-1 (PD-L1) inhibitor atezolizumab and the vascular endothelial growth factor (VEGF) inhibitor (anti-VEGF) bevacizumab is now the preferred first-line standard of care, as recommended in the recently revised American Society of Clinical Oncology guideline [67,89,90,91]. Several ICIs target the PDL1–PD-1 pathway, while VEGF inhibitor therapies reduce VEGF-mediated immunosuppression within the tumor and its microenvironment [67,92]. Other ICIs may activate cytotoxic T-lymphocytes (CTLs) for tumor destruction [92]. A combination of these therapies has the potential for improved efficacy compared with monotherapies by removing immunosuppression and enhancing T-cell infiltration [69,85,93,94]. In HCC, ICIs have shown promising activity when paired with anti-angiogenic agents, other molecularly targeted therapies, and complementary ICIs [85,95,96]. 

**Figure 5:**
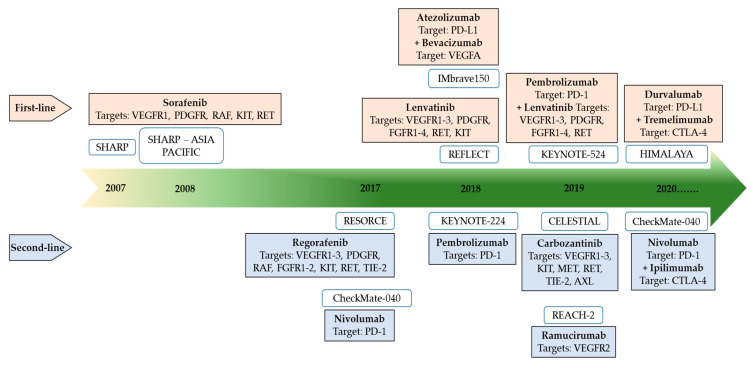
Overview of the key systemic agents approved for management of HCC. Figure adapted from Huang A et al., STTT2021 [87]. AXL; tyrosine protein kinase receptor UFO; CTLA, cytotoxic T-lymphocyte antigen; FGFR, fibroblast growth factor receptor; KIT, proto-oncogene, receptor tyrosine kinase; MET, mesenchymal-epithelial transition factor; PD-L1, programmed cell-death ligand-1; PDGFR, platelet-derived growth factor receptor; RAF, rapidly accelerated fibrosarcoma; RET, rearranged during transfection; TIE-2, tyrosine protein kinase receptor Tie-2; VEGFR, vascular endothelial growth factor receptor.

Patients who are ineligible for treatment with an atezolizumab plus bevacizumab combination regimen receive tyrosine kinase inhibitors (TKIs), sorafenib, lenvatinib, or regorafenib. Sorafenib blocks the proliferation of tumor cells and inhibits angiogenesis by suppressing the RAF/MEK/ERK pathway and by inhibiting VEGF receptors (VEGFR) 2/3, platelet-derived growth factor receptors, fibroblast growth factor receptors, and stem cell factor receptors [97]. In a retrospective study conducted by Rasul et al. assessing the safety and efficacy of sorafenib in patients with advanced HCC and Child-Pugh A/B from the GCC region who failed or were ineligible for local palliative ablation therapy, sorafenib was well tolerated with improved survival, which was more pronounced in the Child-Pugh A group [98]. Similarly, another retrospective study of 150 patients in Qatar showed that the use of sorafenib in HCC patients with preserved liver function resulted in a survival benefit of up to 18 months [15]. Patients with Child-Pugh A had a distinct survival advantage over those with Child-Pugh B and Child-Pugh C due to the least compromised liver function [15]. Current guidelines recommend that treatment with regorafenib after sorafenib failure may lead to an improved OS of up to 26.0 months [99]. Studies demonstrated that treatment with regorafenib yielded a clinical benefit regardless of the last dose of sorafenib or the time-to-progression on sorafenib [99]. Additionally, there are no available and approved systemic therapies for patients with advanced-stage HCC who progress after first-line therapy with sorafenib, possibly due to the strict inclusion criteria for clinical trials. It has been recently reported that metronomic capecitabine had anti-tumor efficacy in patients with advanced-stage HCC and liver cirrhosis (Child-Pugh B-class liver function) who either did not tolerate sorafenib or progressed during sorafenib therapy [100]. Lenvatinib was approved for advanced-stage HCC based on results from the REFLECT trial. Subgroup analyses have shown that lenvatinib is particularly efficacious in Asian patients with underlying HBV infection and patients with high AFP serum concentrations (>200 ng/mL) [5,69,93,94]. The search for more effective treatment of HCC continues, with several trials ongoing that combine an ICI with another agent such as a multiple kinase inhibitor or an anti-angiogenic agent [90] (Figure 5).

### 3.4. Challenges with Established Systemic Therapies in the Gulf and Emergence of Novel Combination Therapies

The differing etiologies of HCC in the Gulf make effective treatment challenging. In a retrospective study of 111 eligible patients with advanced HCC in Kuwait, 39 patients (35%) who received sorafenib showed a median OS of nine months, compared to only one month for those who did not receive sorafenib [13,14]. However, patients with HCV-related HCC who received sorafenib showed a median OS of seven months, while those with HCC of nonviral etiology showed a median OS of 12 months with sorafenib treatment [13,14]. This finding was contrary to results from other studies that have shown that patients with HCV-related HCC had a better response to sorafenib versus those with non-viral etiology and other underlying causes of cirrhosis like HBV infection [13,101]. This variation could be due to the small number of patients involved in the study, a lack of documentation of viral risk factors for HCC in the patient files, and/or an incomplete registry of all HCC cases in the cancer file registry [13]. It is hypothesized that differences in outcomes among HCC patients who received sorafenib were based on their underlying hepatitis viral etiology at presentation [85,93,101,102]. 

TKIs, especially sorafenib, result in AEs that are mostly related to the inhibition of kinases in normal cells. The most common undesirable effects of sorafenib among patients with HCC in the Gulf are diarrhea and skin disorders, which affect nearly 30–40% of patients [13,14,15,17,103]. Hand-foot-skin reaction is also frequently reported, as are milder forms of skin involvement, including rash, alopecia, stomatitis, and erythema multiforme [13,14,15,17,103]. Dermatological toxicities usually respond to topical therapies and/or dose modification without requiring permanent study drug discontinuation [104]. Other reported AEs of sorafenib include fatigue, hypertension, pancreatitis, hypophosphatemia, lymphopenia, neutropenia, and thrombocytopenia [97]. A recent study has demonstrated that improved management of AEs in patients on sorafenib treatment reduced the risk of unnecessary drug discontinuation, thus extending treatment and improving OS [105]. Compared with sorafenib, lenvatinib is associated with higher rates of Grade 3+ hypertension (23% versus 14%), proteinuria (6% versus 2%), and anorexia (5% versus 1%) [85]. Improved management of AEs may optimize the management of TKIs with a cumulative OS advantage [105]. Additionally, a lower discontinuation rate for AEs may lead to better efficacy of subsequent treatments. For example, regorafenib showed improved survival in patients who progressed to sorafenib, possibly due to preserved liver function after treatment with sorafenib [99,105]. 

Patients with unresectable HCC are at particular risk of gastrointestinal hemorrhage due to a high likelihood of portal hypertension that may be exacerbated by treatments that include antiangiogenic agents [79]. Sorafenib can increase the risk of hemorrhage, especially in patients taking warfarin; an increase in the incidence of cardiac events has also been reported [104]. Hemorrhagic events of any grade occurred in 23% of patients in the lenvatinib arm and 15% of patients in the sorafenib arm of the REFLECT trial, and in 9% of patients in the sorafenib arm versus 13% in the placebo arm of the SHARP trial [93,101,102]. The combination of atezolizumab and bevacizumab was the first regimen to improve OS compared with sorafenib [68,93,101,102]. However, bevacizumab, an extensively characterized anti-angiogenic agent used for multiple cancer indications, may increase the risk of hemorrhage, particularly in patients with HCC who are already vulnerable to hemorrhagic events [79,89,106]. Analysis of safety data from the IMbrave150 trial showed that AEs of particular relevance to atezolizumab (hepatitis, rash, hypothyroidism, infusion-related reaction, hyperthyroidism, pancreatitis, and T2D) occurred in 68.7% of patients receiving atezolizumab plus bevacizumab and in 82.1% of patients receiving sorafenib [68]. AEs of particular relevance to bevacizumab, including hemorrhagic events, venous or arterial thromboembolic events, hypertension, and proteinuria, occurred in 57.8% of patients receiving atezolizumab plus bevacizumab and in 48.7% of patients receiving sorafenib [66,89,106]. Clinically significant portal hypertension associated with a high risk of gastro-esophageal varices is common in patients with HCC and was present in 42% of patients with Child-Pugh A and 72% of patients with Child-Pugh B or C liver function [90]. Prospective studies have consistently demonstrated that the risk of variceal hemorrhage is related to the size of the varices [90]. Patients who are very likely to have high-risk varices are those with decompensated cirrhosis, a platelet count ≤150,000/mm^3^, and liver stiffness ≥20 kPa (determined by transient elastography). Thus, several important risks should be considered prior to initiating atezolizumab plus bevacizumab combination therapy in relation to an individual patient’s characteristics. Patients with unresectable HCC often have cirrhosis or portal hypertension (or both) associated with esophageal or gastric varices and portal gastropathy. Portal hypertension can also be due to treatment or portal vein invasion [89]. Therefore, the disease alone can increase the risk of hemorrhage, which in turn may be exacerbated by treatment. Thus, it is recommended that upper gastrointestinal endoscopy be used within 6 months from study entry to assess the risk of hemorrhage from varices in all patients with HCC prior to initiation of atezolizumab plus bevacizumab therapy [67,103,107,108]. This represents a change in practice for the screening of patients in first-line therapy, as upper gastrointestinal endoscopies need to be performed prior to treatment initiation. 

The two hallmark pathologic characteristics of HCC include angiogenesis and immune evasion. While atezolizumab plus bevacizumab is the first-line standard of care for HCC, several combination therapies are being studied to determine the most efficacious subsequent treatment options. Recent studies have demonstrated synergistic effects of ICIs and TKIs, with both showing immunomodulatory effects on the tumor microenvironment [109]. The combination of pembrolizumab and lenvatinib in the KEYNOTE-524/Study 116 trial (NCT03006926) did not show any meaningful improvement over atezolizumab plus bevacizumab [67,90]. Results from the ongoing Phase III LEAP-002 trial (NCT03713593), while not meeting the pre-specified statistical significance for primary endpoints of OS and progression-free survival (PFS), were consistent with the efficacy and safety of the combination of pembrolizumab plus lenvatinib versus lenvatinib alone as a first-line treatment of advanced HCC [90,107,110]. However, the LEAP-002 study results had no impact on clinical practice. Similarly, primary results from the COSMIC-312 trial evaluating the combination of atezolizumab plus cabozantinib versus cabozantinib or sorafenib alone (NCT03755791) did not meet the pre-specified significance levels for the primary endpoints and could not impact clinical practice guidelines for the treatment of HCC [90,107,110,111]. Multiple other regimens based on combinations of kinase inhibitors, anti-VEGF agents, and ICIs are being investigated, including the HIMLAYA trial (NCT03298451) evaluating the novel combination of durvalumab (anti-PD-L1) and tremelimumab (anti-CTL antigen-4 [CTLA-4]) versus sorafenib as a first-line treatment of advanced HCC [108,112]. This is the first combination immunotherapy with anti-PD-L1 and anti-cytotoxic T-lymphocyte–associated antigen 4 (CTLA-4) antibodies that has been successful in Phase III setting [108]. The HIMALAYA trial demonstrated the superiority of durvalumab plus tremelimumab combination (single tremelimumab regular interval durvalumab [STRIDE] regimen) therapy and non-inferiority of durvalumab monotherapy in terms of efficacy and safety versus sorafenib (hazard ratio, 0.78; 95% confidence interval, 0.65–0.92; *p* = 0.0035) as the first-line treatment of patients with unresectable HCC [108,112,113]. The absence of anti-angiogenesis agents in the HIMALAYA treatment regimen reduces the risk of treatment-related bleeding for gastroesophageal varices, with no variceal hemorrhagic events observed, thus eliminating the prerequisite of upper esophageal endoscopy prior to initiation of treatment [108,113]. Primary Phase III results showed grade 3/4 immune-mediated treatment-related AEs, immune-mediated AEs requiring treatment with high-dose steroids, and immune-mediated AEs leading to discontinuation of treatment were minimal and did not raise any concerns about tolerability. A major advantage of the HIMALAYA STRIDE regimen is that it did not show the AEs associated with combination therapies with ICIs plus anti-VEGF/TKI in advanced HCC related to the effect of molecularly targeted agents such as proteinuria, hypertension, ascites, or encephalopathy [113]. There is a lack of information on post-progression treatment after first-line therapy with atezolizumab plus bevacizumab or with the STRIDE regimen. A multidisciplinary approach may be the key to effective patient management and formulating an individualized treatment plan, particularly in those patients who progress after first-line therapy with atezolizumab plus bevacizumab or the STRIDE regimen [114].

## 4. Conclusions

The majority of patients with HCC in the GCC region are diagnosed in the intermediate or advanced stages, with a dismal prognosis. Despite the similarities in clinical services provided to patients with liver disease, there are differences in the availability of treatment regimens and the level of palliative care. Therefore, getting approvals and reimbursements for treatment options could be a way to bridge the gap in implementing the recommended treatment regimens in the region [102]. About 50–60% of the patients diagnosed with HCC qualify for systemic treatment, while the rest receive the best supportive care. The rapid progression of HCC and liver cirrhosis, along with logistical factors like patients’ access to clinical care, patients’ choice, the availability of medical insurance, and a lack of a multidisciplinary approach, were deemed to be the determining factors affecting patient access to systemic therapy in the Gulf.

Although the understanding and management of HCC in the GCC region have changed dramatically over the last decade, HCC still remains a devastating disease that has a ubiquitous and enormous impact on healthcare systems across the Gulf countries. Better designed trials or prospective cohort studies, especially in the post-progression patient population, will enable the development of an evidence-based approach to better control HCC in the GCC region and improve treatment outcomes. Additionally, lifestyle alterations involving modifications in dietary habits, improving physical activity, and refraining from smoking and alcohol consumption may have a significant impact on reducing the burden of HCC among the population of the GCC countries. These changes can also improve the overall health of the individuals and reduce the incidence of other chronic diseases such as diabetes and cardiovascular disease, which are also risk factors for HCC.
